# Cancer Cell Membrane-Coated Nanosuspensions for Enhanced Chemotherapeutic Treatment of Glioma

**DOI:** 10.3390/molecules26165103

**Published:** 2021-08-23

**Authors:** Yueyue Fan, Wenyan Hao, Yuexin Cui, Mengyu Chen, Xiaoyang Chu, Yang Yang, Yuli Wang, Chunsheng Gao

**Affiliations:** 1 College of Pharmacy, Henan University, Kaifeng 475000, China; fanyueyue2020@163.com; 2 State Key Laboratory of Toxicology and Medical Countermeasures, Beijing Institute of Pharmacology and Toxicology, Beijing 100850, China; haohao2626@126.com (W.H.); Cuiyuexinjn@163.com (Y.C.); cmyybx@163.com (M.C.); amms2013@126.com (Y.Y.); 3 Department of Stomatology, Fifth Medical Center of Chinese PLA General Hospital, Beijing 100071, China; Cxy15010248773@163.com

**Keywords:** biomimetic drug delivery system, cancer cell membrane, nanosuspensions, homotypic targeting, glioma

## Abstract

Effective intracerebral delivery is key for glioma treatment. However, the drug delivery system within the brain is largely limited by its own adverse physical and chemical properties, low targeting efficiency, the blood–brain barrier and the blood–brain tumor barrier. Herein, we developed a simple, safe and efficient biomimetic nanosuspension. The C6 cell membrane (CCM) was utilized to camouflaged the 10-hydroxycamptothecin nanosuspension (HCPT-NS) in order to obtain HCPT-NS/CCM. Through the use of immune escape and homotypic binding of the cancer cell membrane, HCPT-NS/CCM was able to penetrate the blood–brain barrier and target tumors. The HCPT-NS is only comprised of drugs, as well as a small amount of stabilizers that are characterized by a simple preparation method and high drug loading. Similarly, the HCPT-NS/CCM is able to achieve targeted treatment of glioma without any ligand modification, which leads it to be stable and efficient. Cellular uptake and in vivo imaging experiments demonstrated that HCPT-NS/CCM is able to effectively cross the blood–brain barrier and was concentrated at the glioma site due to the natural homing pathway. Our results reveal that the glioma cancer cell membrane is able to promote drug transport into the brain and enter the tumor via a homologous targeting mechanism.

## 1. Introduction

Glioma is a common malignant tumor [1]. Unfortunately, due to the presence of the blood–brain barrier (BBB) and the blood–brain tumor barrier (BBTB), most therapeutic drugs are not able to cross these physiological and metabolic barriers into the brain [2,3,4,5]. Methods that can make drugs enter the brain parenchyma through the BBB in order to reduce the systemic toxicity of drugs and play a therapeutic role need to be further researched [6]. The nano-drug delivery system (NDDS) has several advantages, including prolonging drug circulation time and improving drug-specific distribution. There has been significant progress made in the treatment of cancer, as well as other major diseases, and is one of the most promising areas to emerge in drug discovery [7,8,9]. However, the complexity of the drug loading process and nanomaterial synthesis causes poor stability and reproducibility of NDDS, as well as low drug loading, which is not conducive to the clinical transformation of a nanometer drug delivery system [10]. In addition, some NDDS are able to be easily recognized and cleared via the reticuloendothelial system (RES) of the human immune system after the medication is delivered into the blood. Furthermore, the non-specific proteins and other biological macromolecules in the body fluid can spontaneously cover the protein crown formed on the surface of nanoparticles, which alters its surface properties, interfere with the interaction between nanoparticles and biological systems, and accelerate the elimination of NDDS from the immune system [11,12,13]. Significant tumor heterogeneity and intratumoral environment greatly impair the ability of nanomedicines to accumulate through the enhanced permeability and retention effect (EPR effects), which further affects the therapeutic efficacy of the nanomedicine delivery systems [14,15].

In recent years, researchers have attempted to construct biomimetic drug-delivery systems (BDDSs) that were combined with nanoparticles and biomimetic materials. This causes drug nanoparticles to become self-recognizing substances that avoid recognition by the immune system [16,17]. Among the many biomimetic materials, the cell membrane is one of the materials that endows nanoparticles with unique biological properties [18]. By fusion of the membrane on the surface of the nanoparticles, they are able to possess the complex and unique surface physicochemical properties of source cells. Furthermore, the stability of these nanoparticles can be improved by acquiring the characteristics of the membrane [19,20]. Across different cell membrane materials, the surface antigens that are expressed by the cancer cell membrane have the domains and homologous binding proteins that bind to the homologous cells, which allows nanoparticles that are coated on the tumor cell membrane to have tumor-targeting properties [21].

However, the cell membrane-coating strategy has been widely applied on nanostructures, which included PLGA nanoparticles, gold nanoparticles, nanogels, and silica nanoparticles generally have moderate drug loading yields [22,23,24]. Herein, we extended the cell membrane coating technique to nanosuspensions (NS), which are regarded as “pure particles of drug”, with extremely high drug loading yields. Nanosuspension is a carrier-free nanoparticle system that leads to remarkably increased drug solubility and dissolution velocity, which is characterized by simple preparation and good repeatability [25]. By using a small amount of surfactants or polymer materials as stabilizers, the particle size of the drug can be stabilized at the nanometer level, while the surface area of the drug significantly increases [26]. Additionally, the dissolution rate, dissolution degree and oral bioavailability of the drug can be improved, which helps reduce drug dose and avoid adverse drug reactions are caused by high dosage [27,28]. Moreover, its universal applicability and simplicity to the majority of drugs make it possible for mass to all poorly soluble drugs [29].

In this study, the drug nanosuspension was wrapped in a glioma C6 cell membrane in order to explore its feasibility in the treatment of brain glioma. Specifically, through the use of insoluble drug nanocrystallization technology, 10-hydroxycamptothecin (HCPT), the model drug, was prepared into the drug nanosuspension (HCPT-NS) using an acid-base microprecipitation combined with a high-pressure homogenization technique. Then, HCPT-NS and the C6 cell membrane (CCM) were fused to obtain the HCPT-NS/CCM. The HCPT-NS/CCM is able to effectively penetrate the BBB and significantly increase drug accumulation at tumor sites in the mouse model of in situ C6 gliomas that were administered via intravenous injection through the tail, thus providing a unique platform for targeting administration of glioma therapy [30].

## 2. Materials and Methods

Hydroxycamptothecin (10-HCPT) with greater than 98% purity was purchased through the Feng li jing qiu Commerce and Trade Co., Ltd. (Beijing, China). The anti-CD44, anti-CD47, anti-CD31 antibodies were purchased through Abcam (Cambridge, UK). All chemical reagents were of analytical reagent or higher-purity grades and purchased from Macklin Biochemical Co., Ltd. (Shanghai, China).

C6 glioma cells, bEnd.3 brain microvascular endothelial cells, HUVECs human umbilical vein endothelial cells, 4T1 breast cancer cells, B16 melanoma cells and HepG2 hepatocellular carcinoma cells were supplied via the Cell Resource Centre of IBMS (Beijing, China). The cells were cultured in Dulbecco’s modified Eagle’s medium (DMEM), supplemented with 10% FBS (Gibco) and 100 U/mL penicillin. Cells were then stored in an incubator at 37 °C with 5% CO_2_ and passaged every 2 days. The male and female ICR mice (start weight 20 ± 2 g) were obtained from SPF Biotechnology Co., Ltd. (permit number: SCXK (Jing) 2019-0010, Beijing, China) and kept at 20 ± 2 °C. The animals were provided free access to food and water. They were allowed to acclimatize to their environment for at least one week prior to the experiments. All procedures involving care and handling of animals were granted by the Animal Care and Use Ethics Committee of Beijing Institute of Pharmacology and Toxicology (Beijing, China). The animal-related experiments were also approved by this committee.

### 2.1. Preparation of Nanosuspensions

The synthesis of the hydroxycamptothecin nanosuspension is based on a previously reported method of alkali dissolution, as well as acid precipitation combined with high-pressure homogenization. Overall, a total of 50 mg HCPT was accurately weighed and dissolved in 2 mL (0.2 mol·L^−1^) of sodium hydroxide. Under ultrasonic conditions, the 2 mL HCl (0.2 mol·L^−1^) was slowly added in, while being intensely stirred. The acid precipitation process was divided into two parts. After the first step of acid precipitation (adding 90% volume of hydrochloric acid) was carried out, the solution was centrifuged (10,000 rpm·min^−1^, 10 min). Additionally, the previously obtained nanosuspension was separated from the system, and the supernatant was then subjected to the second step of acid precipitation, which required adding the remaining 10% volume of acid, and separation of the nanosuspension. The untransformed carboxylate type was then completely transformed at a low concentration and the influence of salt ions on stability was removed. The precipitate was obtained by combining the two centrifugation solutions. The precipitate was added to the appropriate amount of deionized water, homogenized using a high-pressure homogenizer (D-3L, Kangjinglong, Suzhou, China) (3 cycles at 5 × 10^4^ bar and 25 °C) and 15 mg poloxamer 188 was added, in order to obtain smaller particle size and more stable HCPT nanosuspensions [31].

### 2.2. Preparation and Characterization of C6 Membranes (CCMs)

The cell membranes from C6 cells were prepared according to recent reports in the literature [32]. The C6 cells were cultured in DMEM high glucose medium with 10% FBS (37 °C, 5% CO_2_) for the C6 membrane production. Upon reaching 80−90% confluence was reached, the remaining medium and dead cells were removed by washing with PBS. After digestion with trypsin, cells were centrifuged at 2000 rpm × 2 min in order to collect and wash with PBS. The tumor cells were dispersed in a 25% PBS low-permeability solution that contained protease inhibitors, centrifuged (20,000× *g*·min^−1^) to remove the cancer cell nucleus and other substances, and the cancer cell membrane was obtained using ultra-high-speed centrifugation (100,000× *g*·min^−1^) that was stored in PBS at 4 °C for later use.

### 2.3. Preparation of HCPT-NS/CCM

In order to encapsulate the 10-HCPT nanosuspensions into the C6 membrane in a proper concentration and proportion, we conducted out pre-experiments in order to explore the optimal reaction parameters and the ratio of components within the reaction system. The HCPT-NS solution (1 mL, 1 mg·mL^−1^) was added to the CCM solution (0.5 mL, 0.3 mg·mL^−1^) and then ultrasonically treated in an ice bath (300 W, work 2 s and stop 5 s) for 10 min in order to achieve CCM coating. The mixed solution was centrifuged at 10,000 rpm for 5 min to remove the excess membrane, and the obtained HCPT-NS/CCM was suspended in deionized water for future use.

### 2.4. In Vitro Characterization of Biomimetic Nanomaterials

The particle size and Zeta potential of the newly prepared HCPT-NS and HCPT-NS/CCM were quantified using dynamic light scattering (DLS) (Litesizer™ 500, Anton-Paar, Graz, Austria) analysis. The morphology of these particles was valuated using the transmission electron microscope (TEM) (HITACHI, H-7650, Tokyo, Japan). After proper dilution, they were added to a special copper net with supporting film, respectively. The dye solution was then stained using 3% sodium phosphotungstate solution for 1 min. After it was dried, the morphology was observed under TEM. Particle size was measured using DLS to analyze stability, and measurements were carried out for five consecutive days at 4 °C. The protein components of the C6 cell membrane were determined using polyacrylamide gel electrophoresis and western blot in order to validate retaining the cell membrane surface proteins and successful camouflage of NPs via cancer cell membranes. Overall, 5 mL of HCPT-NS, CCM and HCPT-NS/CCM were taken, centrifuged at 4 °C, at 15,000 rpm·min^−1^, and 10 min in order to collect the precipitate, to which 0.5 mL protein lysate was added after removing the supernatant. The protein concentration was determined using the BCA method. These samples were treated with anti-CD44, anti-CD47, and then anti-mouse IgG secondary antibody conjugated with horseradish peroxidase was further incubated at 37 °C for 1 h, while protein expression was analyzed using gel electrophoresis. The secondary structure of cell membrane proteins has an important role in maintaining the physiological activity of proteins. The circular dichroism (CD, DSM 1000, OLIS, Athens, GA, USA) was utilized to determine changes in the secondary structure of proteins across different preparations in order to further clarify the activity of membrane proteins.

Specific membrane proteins are markers of the functional integrity of the cell membrane. Specific membrane proteins also have a significant role in the interaction and immune escape between tumor cells. CD47 is known to be expressed on the surface of cancer cells, and enhances the ability of immune escape to macrophages. It has generally been considered to be a protective receptor for cancer cells against an attack by the host immune system. Furthermore, CD44 is overexpressed on the surface of malignant tumor cells and is known to be involved in cell-cell interaction, cell adhesion and cell migration. Western Blot analysis was carried out to assess the expression of CD47 and CD44. Hence, the effects of different preparation techniques on the expression of specific proteins remain to be investigated.

### 2.5. In Vitro Release Profile

The in vitro release was examined using the thermostatic oscillation method. HCPT API, HCPT-NS and HCPT-NS/CCM were each 1 mL (HCPT concentration was 1 mg·mL^−1^) and placed in 50 mL of deionized water. The release medium was pH 6.8 and pH 7.4 buffers, containing 0.5% Tween-80, and were selected in order to simulate the environment of body fluids, as well as horizontal shaking at a rotating speed of 100 rpm·min^−1^ at 37 °C ± 1 °C. Overall, 2 mL of release medium was sampled at different time intervals. Next, the same amount of preheated release medium was added. The drug content was then determined using RP-HPLC (1200, Agilent, Santa Clara, CA, USA). After the solution was filtered using a 0.2 μM membrane, drug concentration of HCPT was identified by HPLC, and the cumulative release percentage of drug was calculated.

### 2.6. Homotypic Targeting

In order to verify the ability of HCPT-NS/CCM to target homotypes of cancer cells, we evaluated the uptake of HCPT-NS/CCM across different cells [33]. Thus, 4T1 breast cancer cells, B16 melanoma cells, HepG2 hepatocellular carcinoma cells and C6 brain glioma cells were seeded onto laser confocal dishes (10,000 cells each microplate), respectively. After incubating overnight, the DiI-labeled biomimetic nanosuspension (Free DiI, DiI-HCPT-NS/CCM) was added. After incubating for 0.5 h, the pre-cooled PBS was washed three times, and Hochest 33258 was stained for 10 min. Next, the uptake of different cells was observed using a confocal laser scanning microscopy (CLSM; LSM 880, Zeiss, Oberkochen, Germany).

### 2.7. Cellular Uptake of HCPT-NS/CCM Assay

The cancer cell-targeting ability of biomimetic nanoparticles were assessed with regards to the cellular uptake of HCPT-NS/CCM. In brief, bEnd.3 cells, HUVECs and C6 glioma cells were seeded onto laser confocal microplates at a density of 1 × 10^5^ per microplate and then incubated under standard conditions. After a 24 h incubation period, a different type of DiI-labeled biomimetic nanosuspension was incubated (Free DiI, DiI-HCPT-NS/CCM) at 5% CO_2_ and 37 °C for 30 min. After the incubation, the solution was gently aspirated and washed with PBS three times. The cells were fixed in 4% paraformaldehyde solution for 10 min. This was followed by nuclear staining of the cells with Hochest 33258 dye for 10 min. Finally, cells were sealed with 50% glycerin. The cellular uptake of biomimetic nanoparticles was observed using CLSM, and cellular uptake was quantified using flow cytometry (FCM; FACS Aria III, BD, Piscataway, NJ, USA).

### 2.8. In Vitro Penetration and Targeting Ability of HCPT-NS/CCM on BBB/BBTB Model Assay

We constructed an in vitro BBB model, as previously reported [34]. In brief, the bEnd.3 cells were seeded onto the upper chamber, while C6 cells were placed into the lower chamber at a density of 1:5 of bEnd.3/C6 ratio. The cells were cultured until the confluence reached 100%, during which the transendothelial electrical resistance (TEER) of the cell membrane was recorded. When TEER was over 200 Ω.cm^2^, the culture medium in each upper chamber was altered by different types of DiI-labeled biomimetic nanosuspension (free DiI, DiI- HCPT-NS/CCM). After being cultured for 4 h, the cellular uptake was observed using CLSM. Similarly, HUVECs and C6 cells were utilized to construct a BBTB model to investigate the penetration ability of the preparation.

### 2.9. In Vitro Assessment of Anti-Glioma Efficacy of HCPT-NS/CCM

The cytotoxicity of HCPT, HCPT-NS, and HCPT-NS/CCM on C6 cells was evaluated using the CCK8 assay (*n* = 6). The logarithmic phase C6 cells were seeded onto 96-well plates at a density of 5000 cells per 200 microliters per well. After incubating at 37 °C for 24 h, the medium was removed, and HCPT, HCPT-NS or HCPT-NS/CCM with gradient concentration was added into each group. After incubating the cells for 48 h, 20 μL of CCK-8 solution was added to each well for an additional 2 h. The absorbance of each well was quantified at 450 nm by an ELISA detector OD, and the cell inhibition rate was calculated.

A Transwell chamber (Corning, New York, NY, USA) was utilized to evaluate the inhibitory effect of HCPT on glioma cells in the BBB and BBTB models in vitro. The in vitro BBB and BBTB models were developed in the step above. The culture medium in the apical chamber was replaced utilizing free HCPT and different HCPT formulations when cells were full and the TEER was over 200 Ω.cm^2^, The cells were cultured for 48 h, and anti-tumor effects of various HCPT preparations in vitro BBB and BBTB model were determined via the CCK8 method.

### 2.10. In Vivo Glioma-Targeting Effect and Biodistribution of HCPT-NS/CCM Assay

Firstly, male and female ICR mice were utilized to develop an intracranial glioma-bearing mice model, as previously reported. In brief, C6 cells in the logarithmic phase were taken and digested into a single cell suspension with a cell density of 1 × 10^8^/mL. Next, the mice were fixed onto a brain stereotaxic apparatus with a cell density of 1 × 10^6^ per mouse. The striatum, the site of a high incidence of glioma, was chosen as the location point. The cells were then injected into the ventricle through the meninges and tissues using a micro-syringe. The micro-syringe was moved to the injection hole and then penetrated deep into the brain tissue by inserting the syringe vertically down 4 mm. Then, the needles were withdrawn 1 mm, and then the cells were slowly injected at the speed of 2 μL·min ^−1^. After the cells were injected, the needle was retained, for an extra 1 min, and then finally slowly pull out. After, the wound was sutured and the mice were fed normally [35].

In order to investigate the in vivo tumor accumulation and penetration of the biomimetic nanosuspension DiR, a near-infrared fluorescent dye was efficiently co-loaded onto the HCPT-NS/CCM to form the DiR-HCPT-NS/CCM. The mice were then separated into three groups and injected with 200 μL of normal saline, DiR, and DiR-HCPT-NS/CCM. After intravenous administration in the C6 tumor-bearing mice, in vivo imagers were utilized for analysis at different time points using an excitation wavelength of 748 nm, and an emission wavelength of 780 nm. Mice in the 8 h administration group were euthanized after their tumors were visualized. The major organ tissues (brain, heart, liver, spleen, lungs, and kidneys) were immediately removed for in vitro imaging and analysis using the same method.

The successfully modeled brain glioma mice were injected with normal saline, free DiI, and DiI-HCPT-NS/CCM through the tail vein in order to analyze the tumor distribution. The mice were sacrificed 4 h after administration. Next, the brains were removed and fixed in 4% paraformaldehyde for 24 h in the light and resected. The nuclei were stained using DAPI, which has an excitation wavelength of 358 nm, and an emission wavelength of 461 nm (blue). The distribution of nanosuspension in the brain tissue was observed using inverted fluorescence microscopy.

### 2.11. In Vivo Assessment of Anti-Glioma Efficacy of HCPT-NS/CCM

The mice in situ glioma model were randomly divided into five groups (*n* = 10), while included normal, saline, free HCPT, HCPT-NS and HCPT-NS/CCM, which were injected into the tail vein every day at an HCPT dose of 8 mg·kg^−1^. The survival time of the mice was recorded, and the survival curves were drawn. At the same time, the experimental group was observed for seven days. After administration, the brain glioma was evaluated using magnetic resonance imaging (MRI) (PharmaScan 70T/16, Bruke, Los Angeles, CA, USA). Then, the brain was removed and fixed in 4% paraformaldehyde for 24 h. The tumor tissue was analyzed using HE paraffin section, TUNEL cell apoptosis and apoptosis Caspase 3 and CD31 immunohistochemical detection, in order to observe the damaging effect of different drug groups on tumor tissue.

### 2.12. In Vivo Toxicity Evaluation

In order to identify the potential toxicity of different types of HCPT formulations in vivo, normal mice were randomly divided into four groups. They were administered 200 μL normal saline, free HCPT, HCPT-NS and HCPT-NS/CCM by tail vein every two days for 15 consecutive days at an HCPT dose of 8 mg·kg^−1^. All mice were euthanized one day after the last administration, after which the blood cells and serum biochemical indexes were examined. The heart, liver, spleen, lung, kidney and brain were harvested and stained using the histopathological examination.

### 2.13. Statistical Analysis

Quantitative data are expressed as mean ± standard deviation (SD) unless otherwise indicated. One-way analysis of variance (ANOVA) was utilized to determine the significant differences between different groups. *p* < 0.05 is considered statistically significant.

## 3. Results and Discussion

### 3.1. Preparation and Characterization of HCPT-NS/CCM

**Figure 1:**
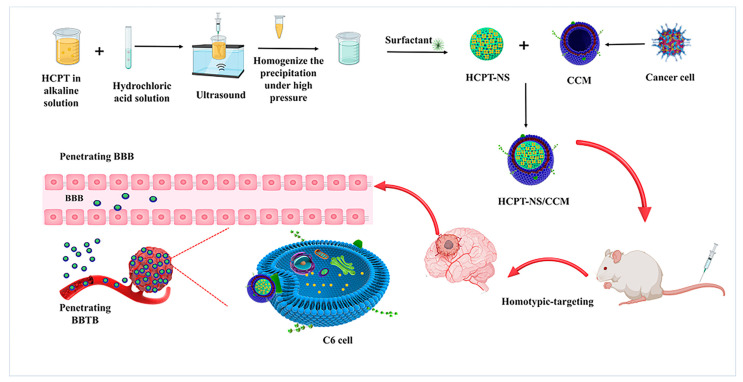
Initially, the HCPT nanosuspensions are prepared using an alkali dissolution, and acid precipitation combined with high-pressure homogenization. Secondly, the cancer cell membrane is separated from the plasma membrane of source cells through the use of hypotonic and differential centrifugation. Then, cancer cell membranes are combined with nanosuspensions, and the mixture is fused in order to construct a biomimetic nanosuspension. Lastly, mice with in situ glioma are treated with an intravenous injection. The biomimetic nanosuspensions penetrated the blood-brain barrier, and the drug was delivered for the treatment of the tumor.

**Figure 2:**
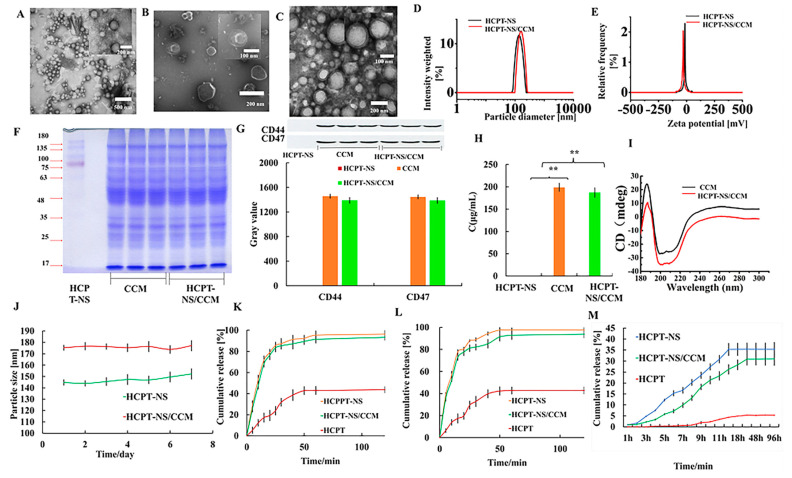
Characterization of HCPT-NS and HCPT-NS/CCM. Transmission electron microscopy images of HCPT-NS (**A**), CCMs (**B**) and HCPT-NS/CCM (**C**). The particle size of HCPT-NS and HCPT-NS/CCM. (**D**) The Zeta potential of HCPT-NS and HCPT-NS/CCM (**E**) The membrane protein of the biomimetic nanosuspensions was assessed by running the protein on an SDS-PAGE gel (**F**) Densitometry analysis of the Western blot (WB) and the relative gray value of membrane-specific proteins CD47 and CD44, *n* = 3 (**G**) Detection of the membrane protein concentration of Biomimetic Nanosuspension via the BCA Method. *n* = 3. (**H**). CD spectra of CCM and HCPT-NS/CCM (**I**). Stability of HCPT-NS and HCPT-NS/CCM in 4 °C. *n* = 3 (**J**) Release kinetics of HCPT under mimicked physiological conditions from HCPT-NS and HCPTNS/CCM at 37 °C in pH 7.4 (**K**), pH 6.8 (**L**) and FBS release medium (**M**), *n* = 3. (The data points represent the mean ± SD, ** indicates *p* < 0.01).

As depicted in Figure 1, HCPT-NS was prepared using an acid-base microprecipitation, combined with a high-pressure homogenization technique, while CCM was prepared using gradient centrifugation. Under the optimal ratio of HCPT-NS to CCM of 3.0, the obtained CCM film was wrapped on HCPT-NS using an ice bath ultrasound. The CCM camouflage was observed via TEM. The HCPT-NS (Figure 2A) was identified as being uniformly spherical. CCM (Figure 2B) presented as a vesicle-like structure, and HCPT-NS/CCM (Figure 2C) was presented as a characteristic core-shell structure. The mean hydrodynamic diameter (Figure 2D) and Zeta potential (Figure 2E) of these nanocarriers were quantified using DLS. The mean hydrodynamic dimeter of HCPT-NS was 145.49 ± 1.85 nm, and the mean Zeta potential was −16.16 ± 0.37 mV. After wrapping the cancer cell membrane, the hydrodynamic diameter and Zeta potential increased and had a mean HCPT-NS/CCM of 174.61 ± 1.38 nm and −30.55 ± 1.07 mV, respectively. The average particle size difference was approximately 10 cm, which is consistent with that of CCM reported in the literature and indicates the successful fusion of CCM and HCPT-NS (Table 1) [36]. The particle size was similar to the results measured by TEM, which further proved the successful preparation of HCPT-NS/CCM.

For further verification, as well as in vivo application, the SDS-PAGE method was utilized to determine the obtained cancer cell membrane. As shown in Figure 2F, CCM and HCPT-NS/CCM have a comparable protein concentration, which demonstrates that the original protein of the cancer cell membrane has basically been retained during the preparation process. Membrane-associated proteins were transferred stably from source cells to CCM and HCPT-NS/CCM. In addition, the cell membrane-specific proteins have a prerequisite for the function of the cell membrane. The cell membrane transmembrane CD47 target protein is able to bind to SIRPα on the surface of macrophages, which can be recognized via the immune system as its own material, and reduce elimination. CD44, as a transmembrane glycoprotein, is widely involved in the heterogeneous adhesion of tumor cells and is also a marker protein of tumor cells. Furthermore, we investigated whether specific proteins that are related to reduced cell adhesion and phagocytic uptake are still expressed in CCM and HCPT-NS/CCM cells. The results of CD47 and CD44 are shown in Figure 2G. The expression of corresponding target proteins in CCM and HCPT-NS /CCM were similar. Gray value analysis has demonstrated that the nanosuspension wrapped by the cell membrane was slightly lost, which was inferred to be a loss caused by the preparation process. These results further indicate that the specific proteins related to decreased adhesion and phagocytosis of C6 tumor cells are still expressed in CCM and HCPT-NS/CCM. Similarly, the BCA method was utilized to quantify the HCPT-NS/CCM protein (Figure 2H). Compared to the prepared cancer cell membrane, protein loss was mainly due to solution transfer and ultrasonic treatment during the preparation process. At the same time, the secondary structure of the protein was scanned using circular dichroism (Figure 2I), and the secondary structure was not destroyed, and the membrane function was retained.

The HCPT-NS and HCPT-NS/CCM depicted good colloidal stability after seven days of storage at 4 °C (Figure 2J). The in vitro release kinetics of each formulation exhibited that the surface energy was larger after nanocrystallization, and nanosuspension significantly improved the drug release rate. HCPT-NS and HCPT-NS/CCM demonstrated similar release behaviors under two different pH release mediums. In pH 7.4 (Figure 2K) and pH 6.8 (Figure 2L), the released chemokine of nanosuspension reached more than 80% in 20 min. The release of HCPT-NS/CCM was slightly delayed, which is likely due to the presence of an outer shell membrane that acts as a diffusion barrier, allowing the drug to be slowly released. The cumulative release rate of HCPT-NS/CCM in fetal bovine serum in 24 h is less than 50% (Figure 2M). This ensures that HCPT-NS/CCM transports the drug to the target site when circulating in the body. At the same time, since the nanoparticle is decomposed in the environment of fetal calf serum for a long time, the cumulative release rate was overall low.

### 3.2. In Vitro Targeting and Efficacy

**Figure 3:**
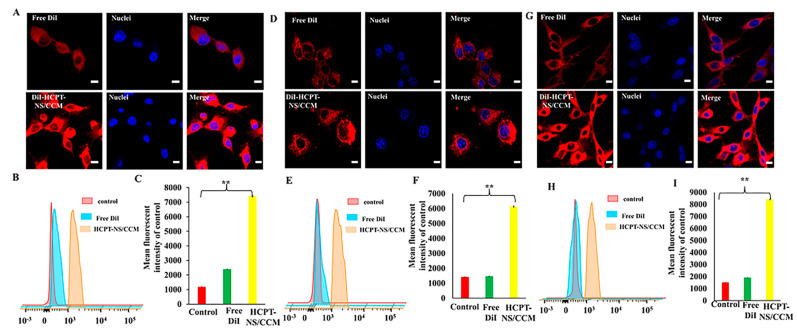
Cellular uptake measured through the CLSM and FCM assays. The confocal images (**A**,**D**,**G**) and the corresponding flow cytometry analysis (**B**,**E**,**H**) of bEnd.3, HUVECs, and C6 cells after exposure to free DiI and DiI-HCPTNS/CCM for 0.5 h at 37 °C at DiI concentration of 10 μg·mL ^−1^, respectively. *n* = 3. Bars represent 20 μm. Image J was utilized to quantify the cellular uptake of free DiI and HCPT-NS/CCM in bEnd.3 (**C**), HUVECs (**F**) and C6 cells (**I**). (The data points represent the mean ± SD. ** indicates *p* < 0.01).

According to literature, the surface adhesion molecules with homologous adhesion domains expressed on cancer cell membrane endow the membrane with the ability of homologous targeting. This unique ability translates to cancer cell membrane-wrapped NPs (CCNPs), which retain the ability to homotypically target primary tumors, as well as metastatic nodules [37,38]. In our homologous targeting verification experiment (Figure S1A), DiI was utilized as a marker in HCPT-NS/CCM, which is a red fluorescent probe for the cell membrane. HCPT-NS/CCM display unprecedented binding and selective uptake in tumor cells, which were matched to those from which they were derived. However, in other types of tumor cells, there was hardly any uptake. In addition, results from cellular uptake of bEnd.3, HUVECs and C6 cells assayed by CLSM and FCM indicate that HCPT-NS/CCM can be significantly taken up by bEnd.3 cells (Figure 3A–C) in comparison to free DiI. This indicates that CCM camouflage is able to enhance uptake of the nanoparticle by brain capillary endothelial cells. The fluorescent intensity of the HCPT-NS/CCM group in HUVECs is also significantly higher than free DiI, which indicates that CCM endows the nanoparticle ability to target tumor neovascular endothelial cells (Figure 3D–F). At the same time, by the homologous targeting mechanisms between tumor cells, HCPT-NS/CCM is able to deliver drugs to the tumor cell, and more drug is taken up by C6 cells (Figure 3G–I). This confirmed that the CCM camouflage can significantly increase the concentration of HCPT in C6 cells.

**Figure 4:**
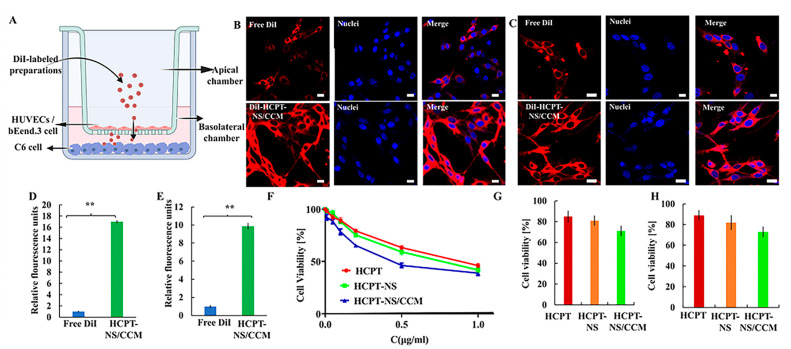
Schematic of the imaging of transwell model (**A**). Transcytosis efficiency of various formulations in the in vitro BBB model (**B**,**D**) and BBTB model (**C**,**E**) measured through the use of CLSM and Plate reader, *n* = 3. Antitumor effect of different HCPT preparations in C6 cells via the CCK8 assay. *n* = 3 (**F**). Antitumor effects of various HCPT preparations crossing the BBB (**G**) and BBTB (H) via the CCK8 assay, *n* = 6. (Bars represent 20 μm, red: DiI, blue: nuclei; The data points represent the mean ± SD. ** indicates *p* < 0.01).

BBB is the main biological barrier that prevents the targeting of nanocarriers, and accumulation in the glioma region. In order to evaluate the BBB permeability of different HCPT preparations in an in vitro transwell model, an in vitro BBB model was successfully developed using the bEnd.3/C6 cells and the resistance value was 260.6 Ω·cm^2^ with the barrier function (Figure 4A). Results indicated that HCPT-NS/CCM had the strongest penetration ability (Figure 4B). Furthermore, the fluorescence intensity of the underlying solution was measured (Figure 4D). HCPT-NS/CCM was found to have the highest brightness, which was significantly different from free DiI, indicating the importance of the cancer cell membrane as a cross BBB camouflage. The BBTB is another major physiological obstacle of drug delivery [39]. BBTB was established by HUVECs/C6 cell co-culture model, with a resistance value of 258.7 Ω·cm^2^. The HCPT-NS/CCM had the strongest targeting ability, similar to the result of BBB (Figure 4C,E).

The CCK-8 method was utilized to investigate the inhibitory effect of free HCPT, HCPT-NS and HCPT-NS/CCM on the proliferation of C6 cells. After 48 h of inhibition, half of the maximum inhibitory concentration (IC_50_) was calculated. The experimental results are demonstrated in Figure 4F. The results showed that each group was able to inhibit the proliferation of C6 cells in a significant concentration-dependent manner, HCPT-NS/CCM had the strongest effect due to its homologous targeting ability, as well as the increased cell uptake, registering an IC_50_ value of 0.4839 μg·mL^−1^, which was significantly lower than HCPT (0.8650 μg·mL^−1^) and HCPT-NS (0.7084 μg·mL^−1^). In addition, the apoptosis rates of C6 cells in BBB and BBTB were measured by the CCK-8 method (Figure 4G,H). The cell survival rates in BBB of HCPT, HCPT-NS and HCPT-NS/CCM were 93.21%, 87.45%, and 67.85%, respectively. The cell survival rates in BBTB were 91.32%, 79.41%, and 60.21%, respectively. The biomimetic nanosuspension had the highest inhibitory effect on the proliferation of cancer cells, indicating that the modification of cancer cell membrane promoted penetration of nano-agents into BBB, BBTB and targeted tumor cells endocytosis.

### 3.3. In Vivo Imaging and Biodistribution

**Figure 5:**
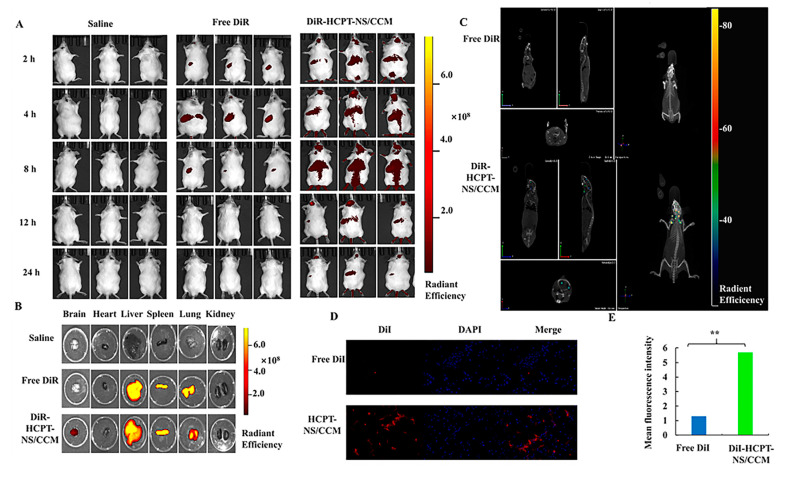
In vivo targeting evaluation. In vivo real-time imaging of saline, DiR-HCPT-NS, and DiR-HCPT-NS/CCM in glioma-bearing mice, *n* = 3 (**A**). Ex vivo fluorescence images of the brain and other major organs of mice treated intravenously with different DiR-encapsulated nanosuspensions (**B**). The brain CT tumor localization scan, which indicates that biomimetic nanosuspensions are able to effectively transport drugs to the brain tissue (**C**). Analysis of the permeability experiment of free DiI and HCPT-NS/CCM on brain tumor site of glioma mice (**D**). Image J was utilized to quantify the distribution of free DiI and HCPT-NS/CCM in brain of mice that bore intracranial C6 glioma (**E**). (DiI: red, DAPI: blue, by 40× object lens; The data points represent the mean ± SD. ** indicates *p* < 0.01).

For effective antitumor therapy, HCPT carriers need to be retained at the tumor site in order to avoid clearance by the body [40]. In vivo imaging is shown in Figure 5A. The fluorescence signal in the brain of tumor-bearing mice in the DiR-HCPT-NS/CCM-treated group was observed at the tumor site 2 h post-injection and fluorescence intensity of DiR peaked at 12 h after injection and remained strongest at 24 h. In contrast, the fluorescence of mice in nanosuspensions without CCM camouflage expressed by free DiR was significantly weak at each time point and then gradually disappeared. These results demonstrate that HCPT-NS has a limited half-life and insufficient tumor accumulation, However, HCPT-NS/CCM has a homotypic targeting ability and exhibits higher tumor accumulation compared to HCPT-NS, This suggests that the cancer cell membrane coating on nanoparticles leads to homogenous (self-identifying) binding to primitive cancer cells. This homogenous affinity lowers systemic clearance, which prolongs blood circulation and effectively improves tumor targeting, which is consistent with our previous results. Quantitative analyses (Figure S1C) demonstrated that the DiR fluorescence intensity of tumor tissue in the DiR-HCPT-NS/CCM-treated group was two folds higher compared to that of free DiR-HCPT-NS treated groups, respectively.

Ex vivo imaging of major organs and tumors further validated these findings. As can be seen from Figure 5B, the brain tissue fluorescence intensity HCPT-NS/CCM biomimetic nanosuspension has the highest concentration and strongest targeting ability in the brain tissues. Among other tissues, the fluorescence intensity was strongest within the liver and spleen. In addition, as the liver is the largest elimination organ, it resulted in the accumulation of biomimetic nanosuspension. The spleen was the largest immune organ, and immune cells capture the biomimetic nanosuspension in the peripheral tissues and transport it to the spleen. Accumulation of HCPT-NS/CCM at the tumor site can be clearly observed utilizing 3D CT scanning fluorescence imaging (Figure 5C), thus verifying the brain targeting ability of HCPT-NS/CCM.

The brain tissues of DiI-labeled biomimetic nanosuspension mice were sliced after injection. As can be seen from Figure 5D, free DiI was not able to enter tumor tissues, and the HCPT-NS/CCM accumulation in tumor tissues was significantly stronger than that of HCPT-NS, which validated its homologous targeting effect and was more conducive to enhancing the targeting effect. Image J chromaticity quantification also demonstrated that HCPT-NS/CCM was most widely distributed within tumor tissues (Figure 5E). A significantly brighter red fluorescence was discovered in HCPT-NS/CCM group, indicating that HCPT-NS /CCM was widely distributed within the tumor and increased tissue penetration, largely due to interaction at the source cell membrane.

### 3.4. Anti-Tumor Effect In Vivo

**Figure 6:**
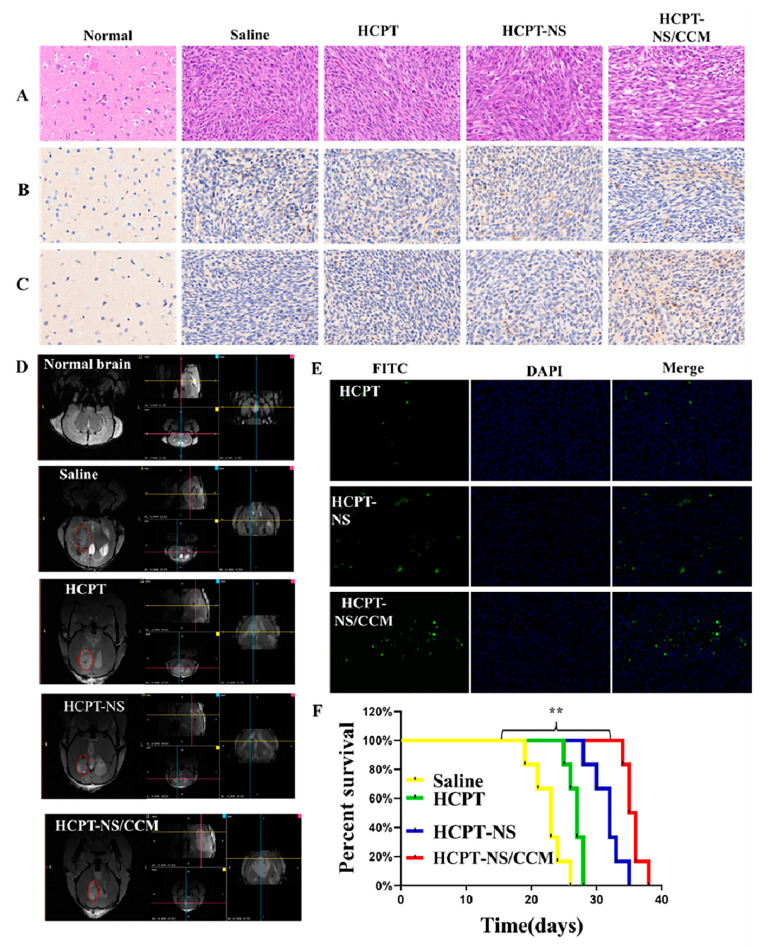
In vivo anti-tumor efficacy on C6 glioma-bearing mice. H&E staining of tumor tissues (**A**). Immunohistochemical staining of Caspase3 (**B**) and CD31 in tumor tissues (**C**). MRI of normal and glioma brains post-treatment (**D**). TUNEL fluorescence detection of brain tumor tissue sections (**E**). Kaplan–Meier survival curves of glioma-bearing mice treated with various HCPT preparations. Dosage: HCPT:8 mg·kg^−1^: The data points represent the mean ± SD (*n* = 10), ** indicates *p* < 0.01 (**F**). (DAPI: blue, FITC: green, Caspase3 and CD31: brown; 20× magnification).

In vivo therapeutic properties of the biomimetic nanosuspension were studied in the glioma-bearing mouse model. H&E staining and histological analysis of the whole brain exhibited that, cancer cells in the saline group exhibited an intact structure, as well as a hyperchromatic nucleus. On the other hand, the tumor tissue in the free-HCPT-treated group and HCPT-NS treated group was sparse. Additionally, tumor cells in mice treated with HCPT-NS/CCM exhibited the sparsest growth, which revealed the highest tumor inhibition efficacy. (Figure 6A). Immunohistochemical (IHC) staining was performed on brain tissues in order to investigate apoptosis (Caspase 3, CC3) and the expression of CD31 receptors related to tumor cell proliferation in the new vessels. Compared to the saline, HCPT and HCPT-NS groups, signals of DNA damage CC3 in the HCPT-NS/CCM group were significantly enhanced and exhibited the highest apoptosis rate (Figure 6B). In addition, CD31 expression in HCPT-NS/CCM group was significantly decreased (Figure 6C). Gadolinium gluconate was utilized as a tumor development enhancer and MRI was used to image brain tumors (Figure 6D). The tumor volume of mice treated with HCPT-NS/CCM was the smallest, which demonstrated a beneficial anti-tumor effect. These results all confirmed the superiority and therapeutic effect of the targeted modified biomimetic nanomaterials. Finally, we performed measurement of the TUNEL tumor tissue apoptosis and fluorescence intensity, which demonstrated that the HCPT-NS/CCM group had the strongest green fluorescence intensity (Figure 6E), indicating that there was significant apoptosis of glioma cells among mice treated with HCPT-NS/CCM. These results were also validated by statistical quantitative results of Image J (Figure S1B). The Kaplan–Meier survival curve (Figure 6F) exhibited that HCPT-NS/CCM significantly extended the lifespan of mice. Importantly, the median survival time (MST) of the HCPT-NS/CCM group was significantly prolonged (35.5 days), in contrast, MST in saline, HCPT and HCPT-NS were 23, 27, and 32 days, respectively. These results demonstrated that HCPT-NS/CCM had the best anti-glioma activity and significant tumor growth inhibition across all groups, which was significantly better compared to HCPT-NS and free HCPT groups due to a higher cellular uptake efficiency, stronger cytotoxicity and greater drug aggregation at the tumor site. HCPT-NS also demonstrated slightly better tumor inhibition than free HCPT but was far less effective than HCPT-NS/CCM, which suggests a weak antitumor effect due to poor targeting ability.

### 3.5. In Vivo Safety

**Figure 7:**
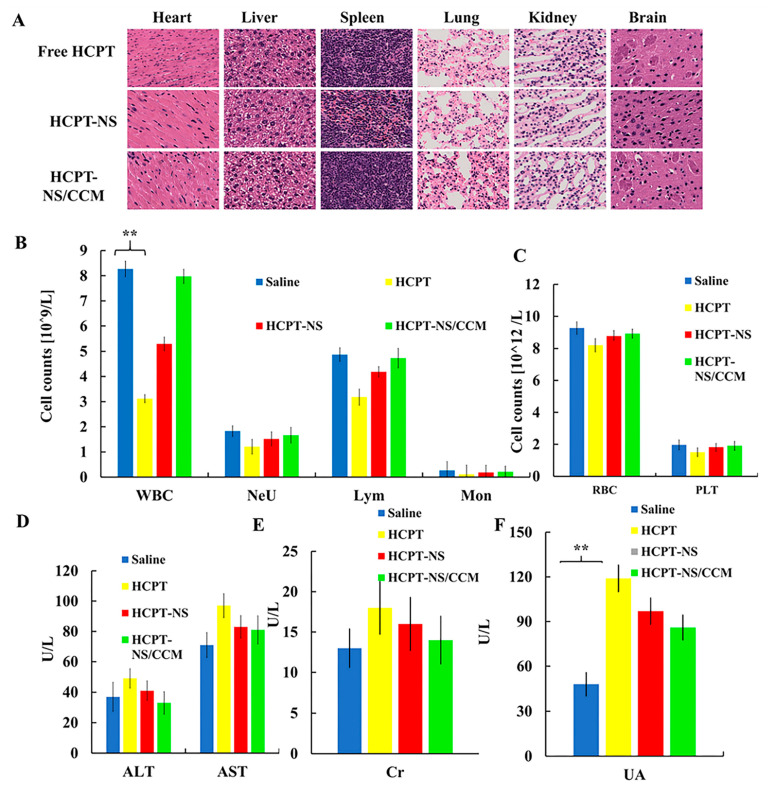
Preliminary safety evaluation. Histopathology of major organs post-treatment. There were no major pathological changes (**A**). Serum biochemical indicators of mice after administration, including counts of immune system cells including WBC, Neu, Lym, and Mon (**B**). Counts of blood cells, including RBC and PLT (**C**). Liver and kidney function markers, including AST, ALT(D), Cr (**E**), and UA (**F**). The data points represent the mean ± SD (*n* = 3). ** indicates *p* < 0.01; 20× magnification.

The biosafety of the biomimetic nanosuspension was assessed using histopathological and hematological analysis. The H&E staining of the tissue in Figure 7A indicated that there was no obvious cell necrosis or inflammation was found in the mouse tissues after HCPT, HCPT-NS and HCPT-NS/CCM. Compared to the other groups, the hematological indicators of mice treated with HCPT-NS/CCM were relatively stable. The white blood cell counts of HCPT treated mice were significantly reduced, which indicated bone marrow suppression, one of the major side effects of HCPT. In contrast, mice treated with HCPT-NS/CCM had fairly stable levels of WBC during treatment, which indicates that nanoparticle encapsulation and cell membrane camouflage can effectively inhibit the toxic side effect. There were no differences in other blood parameters between these groups. In addition, aspartic acid aminotransferase (AST), alanine aminotransferase (ALT), creatinine (CR) and uric acid (UA) in the blood (Figure 7B–F) shows that HCPT can cause mild liver and kidney toxicity. Encapsulation of cancer cell membranes is able to improve hydroxycamptothecin-induced mild liver and kidney toxicity. Thus, these results validate that HCPT-NS/CCM had relatively little effect on the liver and kidney function of the mice. We have shown that HCPT-NS/CCM has excellent biocompatibility in organisms, does not cause side effects to normal tissues, and can be safely applied in biological experiments.

## 4. Conclusions

Currently, cell membrane encapsulation technology is an active field in nanobiomedical research. The combination of natural cell membrane characteristics and nanoparticle characteristics through the use of cell membrane encapsulated nanoparticles not only increases biocompatibility but also achieves better targeting and cycling characteristics in vivo. In this study, we initially sought to improve the low polarity, insoluble in water, poor oral absorption and low bioavailability of hydroxycamptothecin. Thus, HCPT-NS was prepared using the alkali dissolution and acid precipitation in combination with high-pressure homogenization, which significantly improved the saturated solubility and dissolution rate of hydroxycamptothecin, which is characterized by small particle size, high drug loading and easy industrial production. Secondly, HCPT-NS/CCM was prepared through the use of ultrasonic coating method to overcome shortcomings of poor targeting and easy removal in vivo, as well as to endow nanoparticles with the ability to cross BBB and BBTB. The homologous targeting biomimetic nanoparticle delivery system was constructed to better retain adhesion protein on the surface of cancer cell membranes. The results demonstrated that HCPT-NS/CCM was spherical, with a core-shell structure. With a homologous targeting mechanism of the cancer cell membrane and immune escape ability, HCPT-NS/CCM have a good targeting ability for glioma, long blood circulation time, significant inhibition of tumor growth in vivo and in vitro, and prolong the survival time of glioma-bearing mice. In short, due to its excellent homology targeting, long blood circulation time and immune escape ability, tumor cell membrane camouflage nanoparticles demonstrate great potential in tumor treatment and can be easily extended to the delivery of many anticancer drugs.

## Figures and Tables

**Table 1:** Characterization of different formulations.

Formulation	Size (nm)	PDI	Zeta-Potential (mV)
HCPT-NS	145.49 ± 1.85	0.069 ± 0.018	−16.16 ± 0.37
HCPT-NS/CCM	174.61 ± 1.38	0.094 ± 0.015	−30.55 ± 1.07

## Data Availability

Not applicable.

## Supplementary Materials

The following are available online. Figure S1 Cellular uptake of HCPT-NS/CCCM in different kinds of cancer cell measured using CLSM assays (A). The TUNEL tumor tissue fluorescence intensity measurement of Image J; 20× magnification (B). Brain fluorescence intensity analysis of saline, DiR-HCPT-NS, and DiR-HCPT-NS/CCM in glioma-bearing mice (C).

## Sample Availability

Samples of the compounds are not available from the authors.

## References

[1] Wang X., Zhao Y., Dong S., Lee R.J., Yang D., Zhang H., Teng L. (2019). Cell-Penetrating Peptide and Transferrin Co-Modified Liposomes for Targeted Therapy of Glioma. Molecules.

[2] Reddy S., Tatiparti K., Sau S., Iyer A.K. (2021). Recent advances in nano delivery systems for blood-brain barrier (BBB) penetration and targeting of brain tumors. Drug Discov. Today.

[3] Chen C., Duan Z., Yuan Y., Li R., Pang L., Liang J., Xu X., Wang J. (2017). Peptide-22 and Cyclic RGD Functionalized Liposomes for Glioma Targeting Drug Delivery Overcoming BBB and BBTB. ACS Appl. Mater. Interfaces.

[4] Li J., Zhao J., Tan T., Liu M., Zeng Z., Zeng Y., Zhang L., Fu C., Chen D., Xie T. (2020). Nanoparticle Drug Delivery System for Glioma and Its Efficacy Improvement Strategies: A Comprehensive Review. Int. J. Nanomed..

[5] Gao Y., Wang R., Zhao L., Liu A. (2021). Natural polymeric nanocarriers in malignant glioma drug delivery and targeting. J. Drug Target..

[6] Ruan S., Zhou Y., Jiang X., Gao H. (2021). Rethinking CRITID Procedure of Brain Targeting Drug Delivery: Circulation, Blood Brain Barrier Recognition, Intracellular Transport, Diseased Cell Targeting, Internalization, and Drug Release. Adv. Sci..

[7] Liao W., Fan S., Zheng Y., Liao S., Xiong Y., Li Y., Liu J. (2019). Recent Advances on Glioblastoma Multiforme and Nano-drug Carriers: A Review. Curr. Med. Chem..

[8] Moradi Kashkooli F., Soltani M., Souri M. (2020). Controlled Anti-Cancer Drug Release through Advanced Nano-Drug Delivery Systems: Static and Dynamic Targeting Strategies. J Control Release..

[9] Li X., Tsibouklis J., Weng T., Zhang B., Yin G., Feng G., Cui Y., Savina I.N., Mikhalovska L.I., Sandeman S.R. (2017). Nano carriers for drug transport across the blood-brain barrier. J. Drug Target..

[10] Liu D., Yang F., Xiong F., Gu N. (2016). The Smart Drug Delivery System and Its Clinical Potential. Theranostics.

[11] Ganoth A., Merimi K.C., Peer D. (2015). Overcoming multidrug resistance with nanomedicines. Expert Opin. Drug Deliv..

[12] Ye M., Han Y., Tang J., Piao Y., Liu X., Zhou Z., Gao J., Rao J., Shen Y. (2017). A Tumor-Specific Cascade Amplification Drug Release Nanoparticle for Overcoming Multidrug Resistance in Cancers. Adv. Mater..

[13] Auría-Soro C., Nesma T., Juanes-Velasco P., Landeira-Viñuela A., Fidalgo-Gomez H., Acebes-Fernandez V., Gongora R., Almendral Parra M.J., Manzano-Roman R., Fuentes M. (2019). Interactions of Nanoparticles and Biosystems: Microenvironment of Nanoparticles and Biomolecules in Nanomedicine. Nanomaterials.

[14] Danhier F. (2016). To exploit the tumor microenvironment: Since the EPR effect fails in the clinic, what is the future of nanomedicine?. J. Control. Release..

[15] Park J., Choi Y., Chang H., Um W., Ryu J.H., Kwon I.C. (2019). Alliance with EPR Effect: Combined Strategies to Improve the EPR Effect in the Tumor Microenvironment. Theranostics.

[16] Chen Y.X., Wei C.X., Lyu Y.Q., Chen H.Z., Jiang G., Gao X.L. (2020). Biomimetic drug-delivery systems for the management of brain diseases. Biomater. Sci..

[17] Li B., Wang F., Gui L., He Q., Yao Y., Chen H. (2018). The potential of biomimetic nanoparticles for tumor-targeted drug delivery. Nanomedicine.

[18] Zhu C., Ma J., Ji Z., Shen J., Wang Q. (2021). Recent Advances of Cell Membrane Coated Nanoparticles in Treating Cardiovascular Disorders. Molecules.

[19] Chai Z., Ran D., Lu L., Zhan C., Ruan H., Hu X., Xie C., Jiang K., Li J., Zhou J. (2019). Ligand-Modified Cell Membrane Enables the Targeted Delivery of Drug Nanocrystals to Glioma. ACS Nano.

[20] Gao W., Zhang L. (2015). Coating nanoparticles with cell membranes for targeted drug delivery. J. Drug Target..

[21] Wang H., Liu Y., He R., Xu D., Zang J., Weeranoppanant N., Dong H., Li Y. (2020). Cell membrane biomimetic nanoparticles for inflammation and cancer targeting in drug delivery. Biomater. Sci..

[22] Wang R., Yang H., Fu R., Su Y., Lin X., Jin X., Du W., Shan X., Huang G. (2020). Biomimetic Upconversion Nanoparticles and Gold Nanoparticles for Novel Simultaneous Dual-Modal Imaging-Guided Photothermal Therapy of Cancer. Cancers.

[23] Li D., Cui R., Xu S., Liu Y. (2020). Synergism of cisplatin-oleanolic acid co-loaded hybrid nanoparticles on gastric carcinoma cells for enhanced apoptosis and reversed multidrug resistance. Drug Deliv..

[24] Zinger A., Sushnitha M., Naoi T., Baudo G., De Rosa E., Chang J., Tasciotti E., Taraballi F. (2021). Enhancing Inflammation Targeting Using Tunable Leukocyte-Based Biomimetic Nanoparticles. ACS Nano.

[25] Du J., Li X., Zhao H., Zhou Y., Wang L., Tian S., Wang Y. (2015). Nanosuspensions of poorly water-soluble drugs prepared by bottom-up technologies. Int. J. Pharm..

[26] Guo C., Chen Y., Zhu J., Wang J., Xu Y., Luan H., Wang H. (2018). Optimization of Extended-Release ZL-004 Nanosuspensions for In Vivo Pharmacokinetic Study to Enhance Low Solubility and Compliance. Molecules.

[27] Jacob S., Nair A.B., Shah J. (2020). Emerging role of nanosuspensions in drug delivery systems. Biomater. Res..

[28] Rabinow B.E. (2004). Nanosuspensions in drug delivery. Nat. Rev. Drug Discov..

[29] Singh S.K., Vaidya Y., Gulati M., Bhattacharya S., Garg V., Pandey N.K. (2016). Nanosuspension: Principles, Perspectives and Practices. Curr. Drug Deliv..

[30] Zhang L., Zhang X., Lu G., Li F., Bao W., Song C., Wei W., Ma G. (2019). Cell Membrane Camouflaged Hydrophobic Drug Nanoflake Sandwiched with Photosensitizer for Orchestration of Chemo-Photothermal Combination Therapy. Small.

[31] Ao H., Li Y., Li H., Wang Y., Han M., Guo Y., Shi R., Yue F., Wang X. (2020). Preparation of hydroxy genkwanin nanosuspensions and their enhanced antitumor efficacy against breast cancer. Drug Deliv..

[32] Suski J.M., Lebiedzinska M., Wojtala A., Duszynski J., Giorgi C., Pinton P., Wieckowski M.R. (2014). Isolation of plasma membrane-associated membranes from rat liver. Nat. Protoc..

[33] Wu P., Yin D., Liu J., Zhou H., Guo M., Liu J., Liu Y., Wang X., Liu Y., Chen C. (2019). Cell membrane based biomimetic nanocomposites for targeted therapy of drug resistant EGFR-mutated lung cancer. Nanoscale.

[34] Xue J., Zhao Z., Zhang L., Xue L., Shen S., Wen Y., Wei Z., Wang L., Kong L., Sun H. (2017). Neutrophil-mediated anticancer drug delivery for suppression of postoperative malignant glioma recurrence. Nat. Nanotechnol..

[35] Cui Y., Sun J., Hao W., Chen M., Wang Y., Xu F., Gao C. (2020). Dual-Target Peptide-Modified Erythrocyte Membrane-Enveloped PLGA Nanoparticles for the Treatment of Glioma. Front. Oncol..

[36] Chen Z., Zhao P., Luo Z., Zheng M., Tian H., Gong P., Gao G., Pan H., Liu L., Ma A. (2016). Cancer Cell Membrane-Biomimetic Nanoparticles for Homologous-Targeting Dual-Modal Imaging and Photothermal Therapy. ACS Nano.

[37] Harris J.C., Scully M.A., Day E.S. (2019). Cancer Cell Membrane-Coated Nanoparticles for Cancer Management. Cancers.

[38] Jin J., Krishnamachary B., Barnett J.D., Chatterjee S., Chang D., Mironchik Y., Wildes F., Jaffee E.M., Nimmagadda S., Bhujwalla Z.M. (2019). Human Cancer Cell Membrane-Coated Biomimetic Nanoparticles Reduce Fibroblast-Mediated Invasion and Metastasis and Induce T-Cells. ACS Appl. Mater. Interfaces.

[39] Chen L., Zeng D., Xu N., Li C., Zhang W., Zhu X., Gao Y., Chen P.R., Lin J. (2019). Blood-Brain Barrier- and Blood-Brain Tumor Barrier-Penetrating Peptide-Derived Targeted Therapeutics for Glioma and Malignant Tumor Brain Metastases. ACS Appl. Mater. Interfaces.

[40] Jia Y., Sheng Z., Hu D., Yan F., Zhu M., Gao G., Wang P., Liu X., Wang X., Zheng H. (2018). Highly penetrative liposome nanomedicine generated by a biomimetic strategy for enhanced cancer chemotherapy. Biomater. Sci..

