# Supplementary material for: Cancer Cell Membrane-Coated Nanosuspensions for Enhanced Chemotherapeutic Treatment of Glioma
Source: Molecules. 2021 Aug 23;26(16):5103. doi: 10.3390/molecules26165103 (PMC8400986; doi:10.3390/molecules26165103)
Supplement: Supplementary file 1 [file molecules-26-05103-s001.zip › molecules-1309041-supplementary.pdf]

Supplementary Figures：

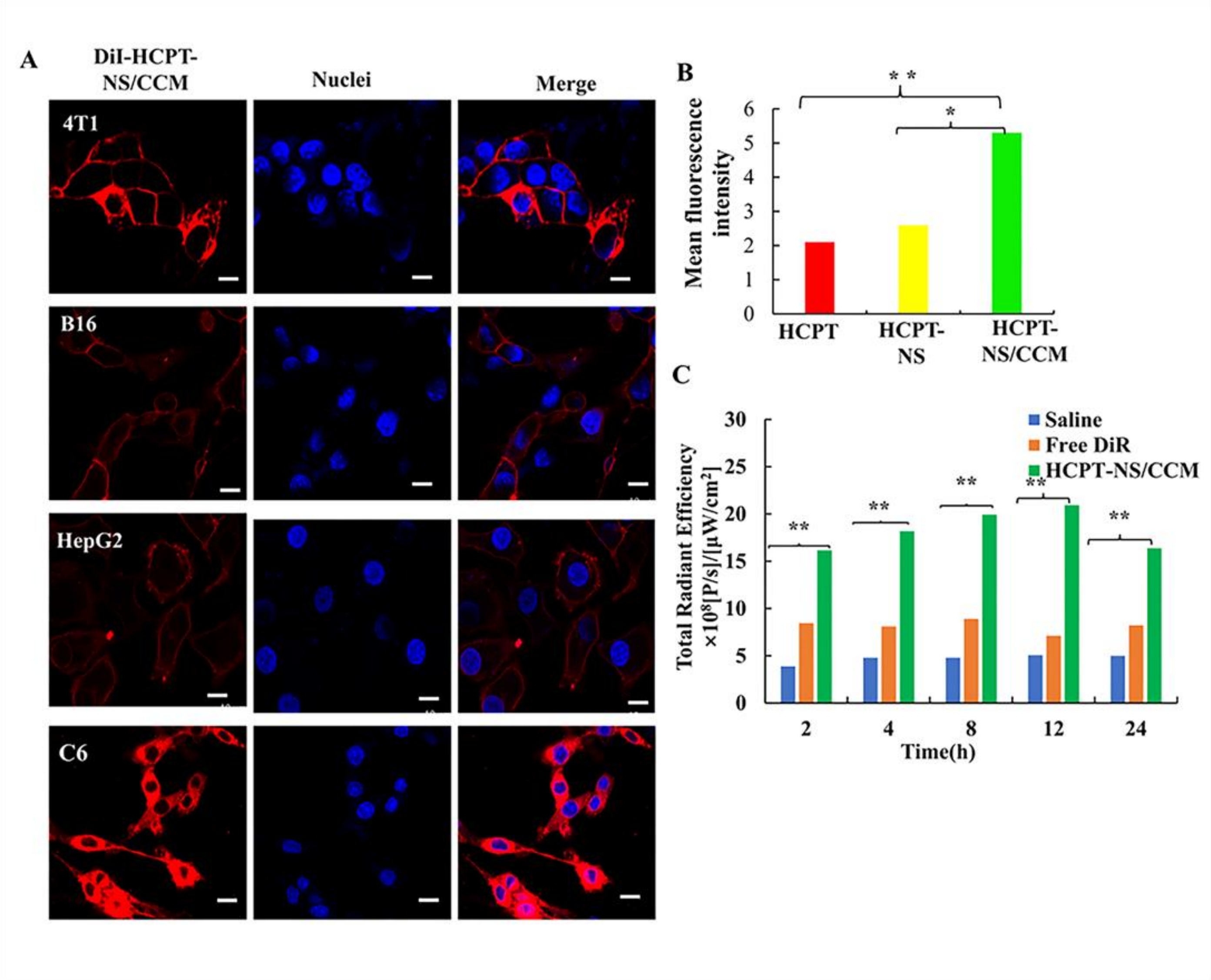

Figure. S1. Cellular uptake of HCPT- NS/CCCM in different kinds of cancer cell measured using CLSM assays(**A**). The TUNEL tumor tissue fluorescence intensity measurement of Image J; 20x magnification(**B**). Brain fluorescence intensity analysis of saline, DiR-HCPT-NS, and DiR-HCPT-NS/CCM in glioma-bearing mice(**C**). * indicates $p < 0.05$; ** indicates $p < 0.01$.
